# Author Correction: INSERT-seq enables high-resolution mapping of genomically integrated DNA using Nanopore sequencing

**DOI:** 10.1186/s13059-023-02979-w

**Published:** 2023-06-09

**Authors:** Dimitrije Ivančić, Júlia Mir-Pedrol, Jessica Jaraba-Wallace, Núria Rafel, Avencia Sanchez-Mejias, Marc Güell

**Affiliations:** 1 Departament de Medicina i Ciències de La Vida (MELIS), Universitat Pompeu Fabra, Barcelona, Spain; 2 The Barcelona Institute of Science and Technology, Barcelona, Spain

**Author Correction: Genome Biol 23, 227 (2022)**

**https://doi.org/10.1186/s13059-022-02778-9**

Following publication of the original article [1], the authors identified an error in Additional file 1: Supplementary figures 1-11 and INSERT-seq protocol. The protocol was not correctly incorporated into the file. The corrected Additional file 1 is given in this correction article and the original article [1] has been corrected.

## Supplementary Information

**Additional file 1.** Supplementary figures 1-11 and INSERT-seq protocol.
