# Supplementary material for: INSERT-seq enables high-resolution mapping of genomically integrated DNA using Nanopore sequencing
Source: Genome Biol. 2022 Oct 25;23:227. doi: 10.1186/s13059-022-02778-9 (PMC9594898; doi:10.1186/s13059-022-02778-9)
Supplement: Supplementary file 1 — Additional file 1: Supplementary figures 1-11 and INSERT-seq protocol. [file 13059_2022_2778_MOESM1_ESM.pdf]

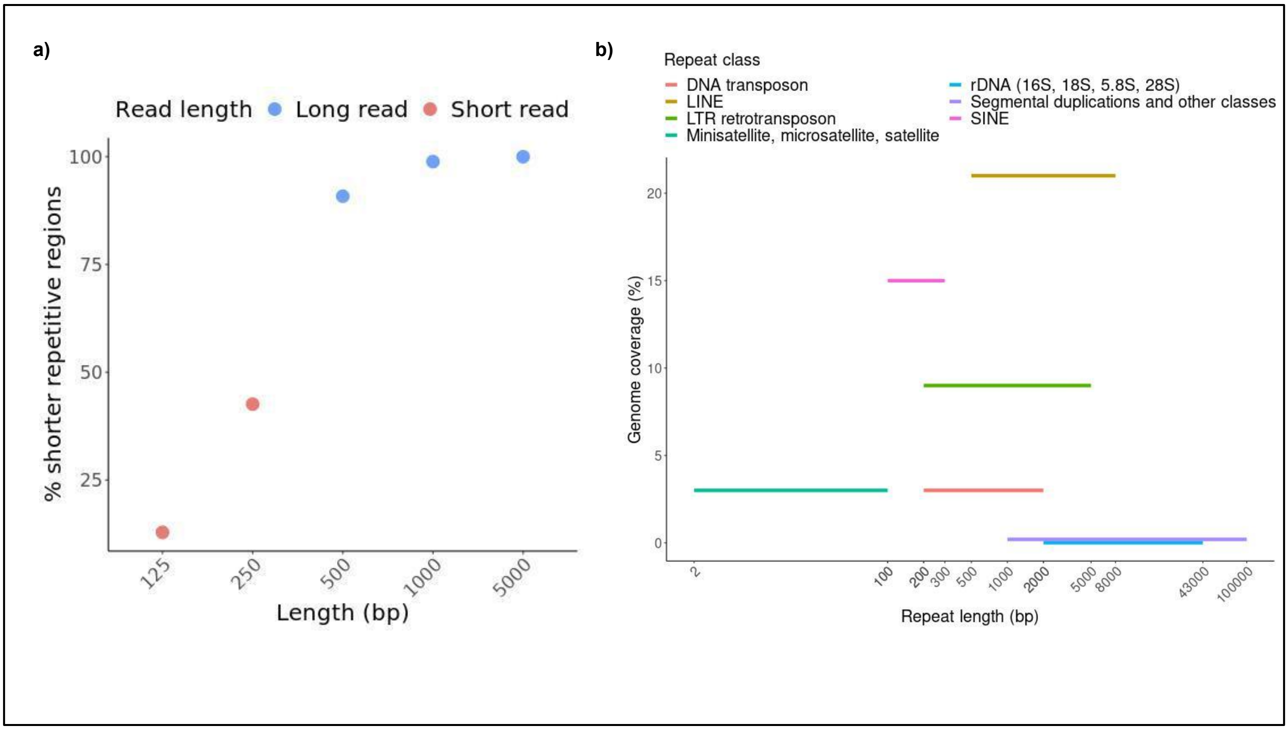

Fig S1 | a) Percentage of Dfam database human genome repetitive regions shorter than 125, 250, 500, 1000 and 5000 base pairs. **b)** Representation of repetitive regions from Human genomes based on their length range in the x axis and the percentage of representation in the genome in the y axis.

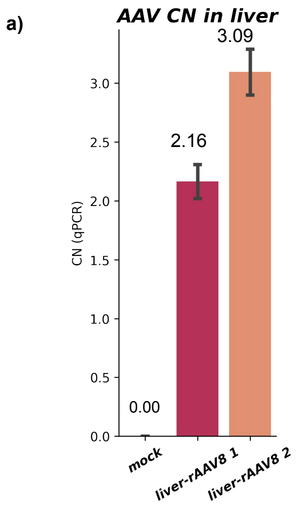

Fig S2 | CN determination a) Quantification of AAV DNA levels in mouse liver by qPCR. Numbers indicate two different animal replicates.

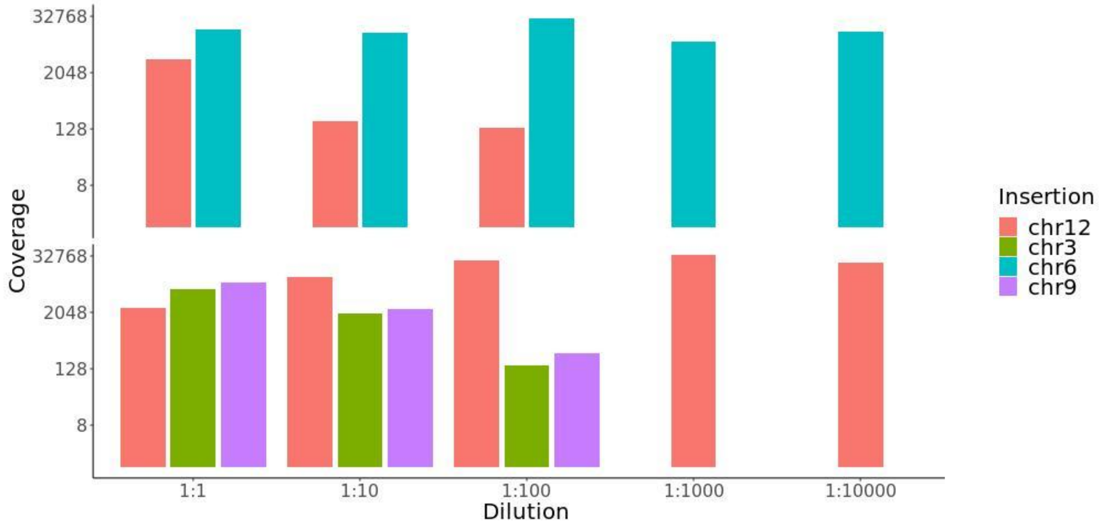

Fig S3| Limit of Detection (LOD) calculation. Insertion detection depending on the sample dilution. Shows the coverage of on-target insertions. On the top panel, a monoclonal cell line with one insertion at chr 12 (MN2 in red) was diluted with a constant amount of a different monoclonal cell line (MN7 with an insertion at chr 6, in blue) in different proportions (1:1, 1:100, 1:1000 and 1:10000). On the bottom panel, MN2 with one insertion at chr 12 (red) was used as a reference and a different monoclonal cell line (MN10 with insertions at chr3 in green and chr 9 in purple) was diluted in different proportions. In both experiments, the limit of detection is at dilution 1:100.

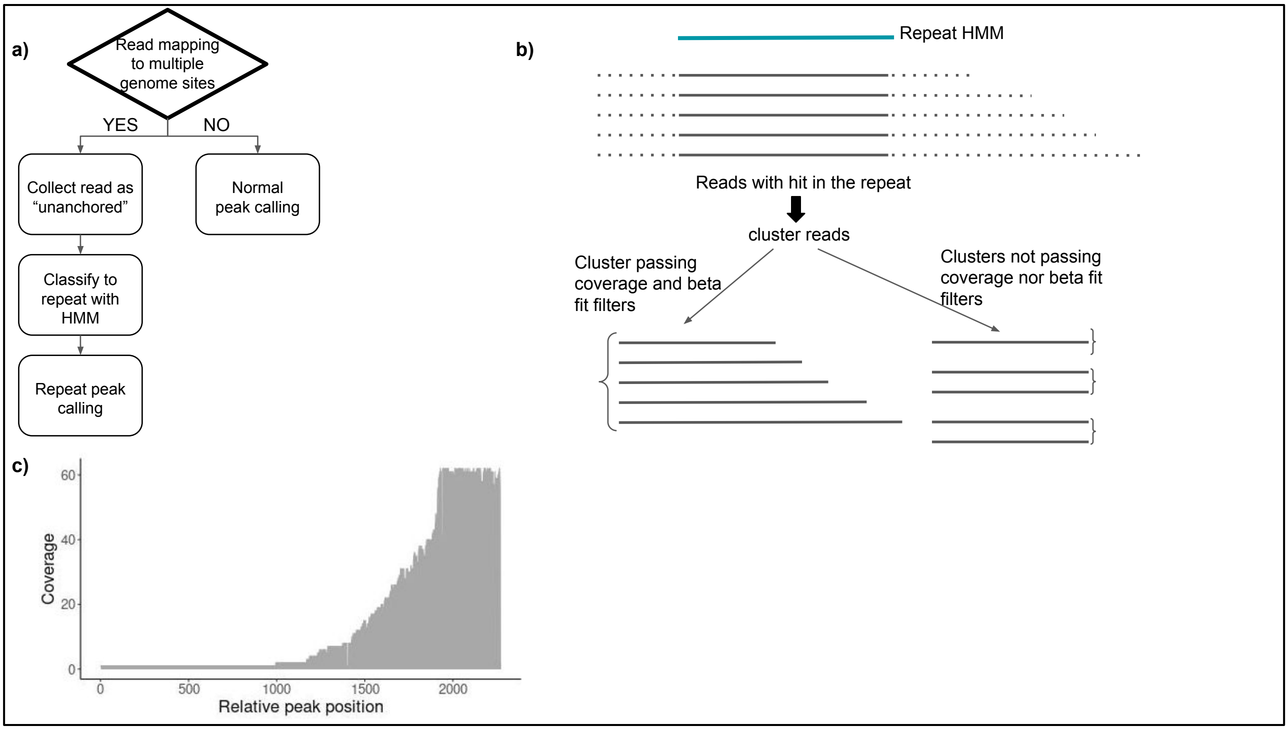

Fig S4 | Unanchored peak calling pipeline. a) Schematic representation of the detection of insertions at repetitive regions. Reads that map to multiple genome sites are collected and mapped to the reference human repeats. A peak calling of the repeats is performed. **b)** Schematic representation of the peak calling of unanchored reads. Unanchored reads are classified to a repeat based on HMM hits, reads from each peak are clustered in order to determine if all the reads belong to the same peak, finally, a coverage filter and standard deviation filters are applied in order to determine if a peak is called. **c)** Peak called at HERVK11 repeat in the clonal test cell line containing multiple lentiviral insertions (MOPO). Coverage of a multiple sequence alignment of all reads that have a hit against the HERVK11 repeat and cluster together.

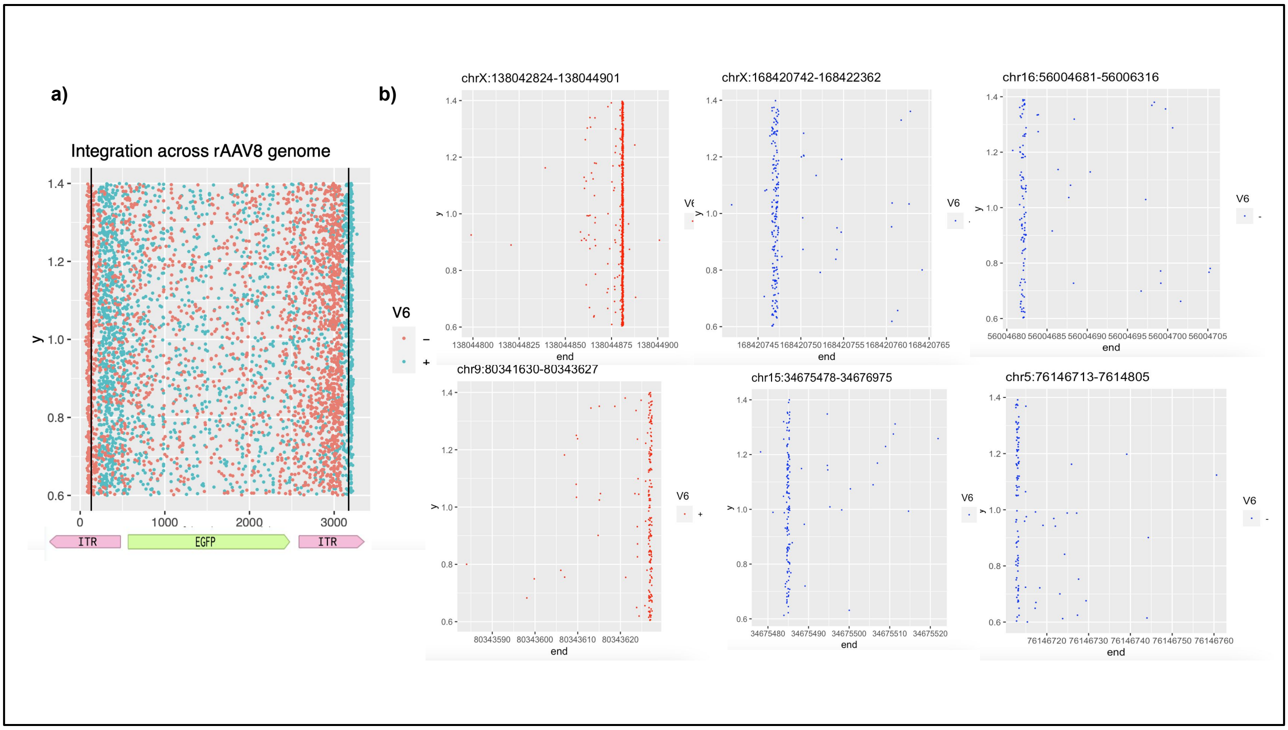

Fig S5 | a) Auto-integration of rAAVs in ITR sequences. Consistent with previous reports, High levels of auto-integrated AAV genomes are detected with INSERTseq. **b)** Integration patterns at most abundant hotspots across Mouse genome (mm10)

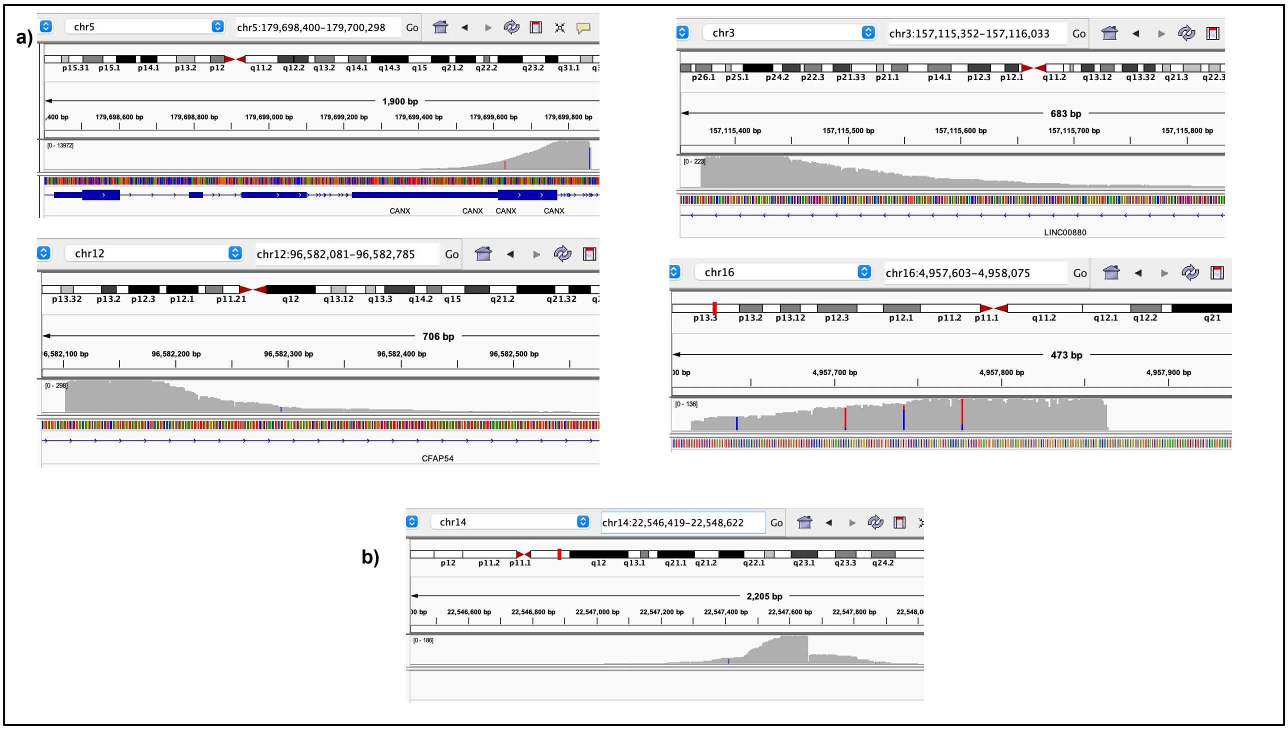

Fig S6 | Integration profile of Cas9-PiggyBac chimeric protein: Coverage at 5 most represented integration sites at **a)** off-target insertions and **b)** on-target insertion. Off-targets in such abundance but occurring at the same location could be originated from clonal expansions.

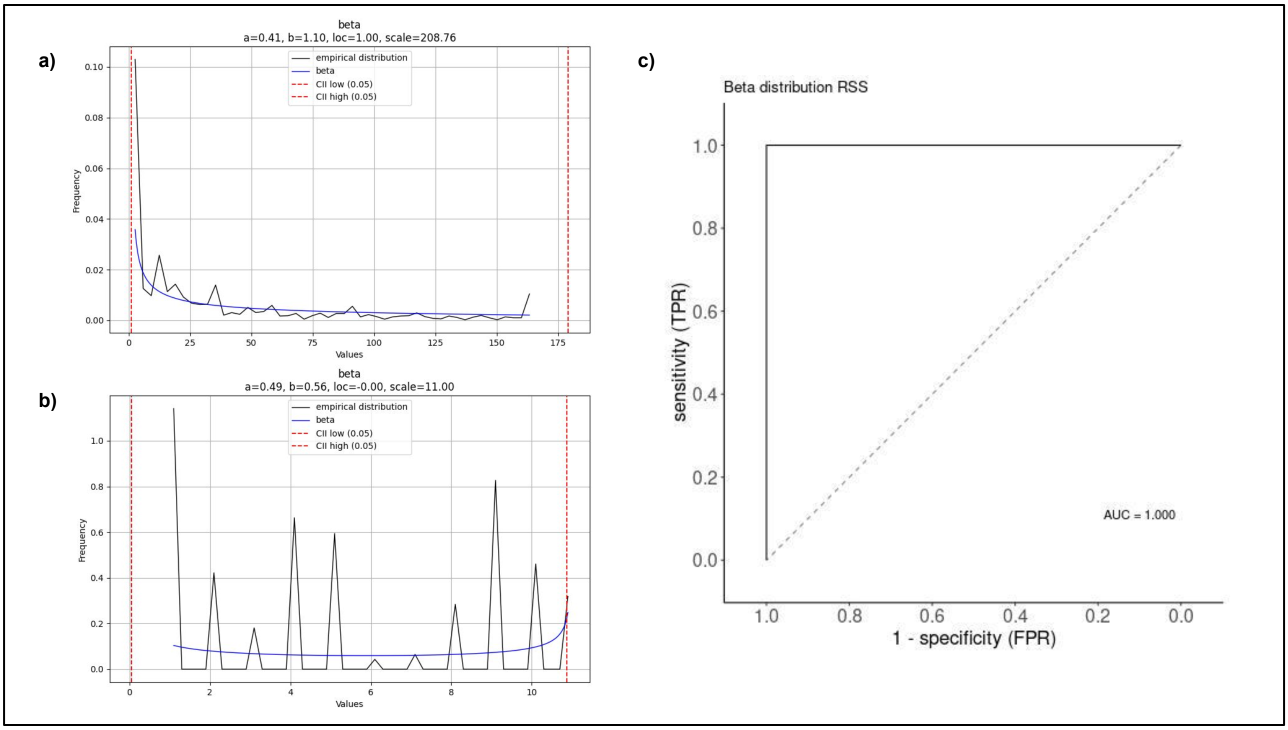

Fig S7 | Peak calling ROC curve of MOPO sample. a) Representation of the distribution of coverage (black) of a true peak from the MOPO sample at chr15:99327982-99330675 fitted to a beta distribution (blue) with RSS=0.005045. **b)** Representation of the distribution of coverage (black) of a false peak from the MOPO sample at chr3:99327982-99330675 fitted to a beta distribution (blue) with RSS=2.831412. **c)** ROC curve of the Residual Sum of Squares (RSS) of peak coverage fit to a beta distribution. The selected threshold was 1.

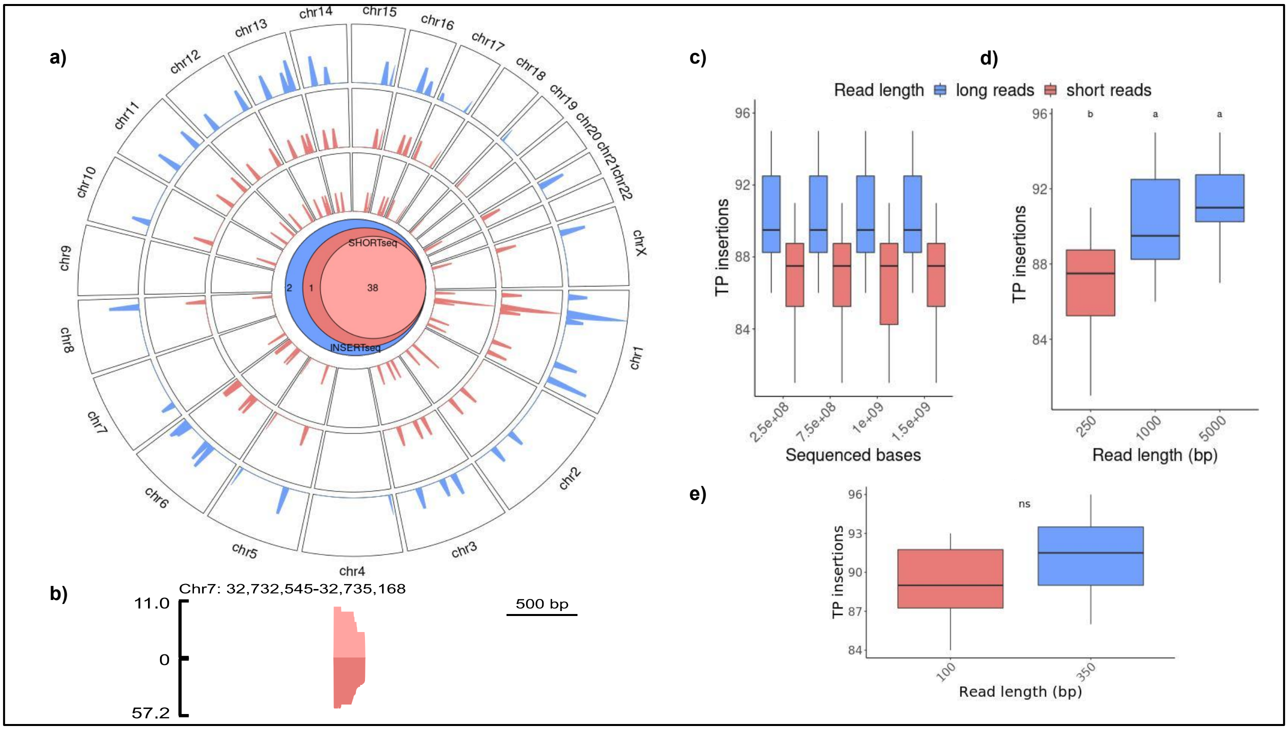

Fig S8 | Effect of increased number of reads. a) Genome-wide map displaying the overlap between insertions detected in MOPO with either short read insertion detection (red) and long read insertion detection (blue). The Venn diagram summarises the overlap of common insertions with the two methods. A higher number of reads was used for a SHORTsesq analysis (dark red) where one new insertion is found respect to the experiment with a subset of reads. **b)** Coverage at insertion site in the mono-clonal poli-insertional (MOPO) analyzed cell line, with a subset of short reads (light red) and the complete run of short reads (dark red). The insertion shown was detected with a higher sequencing depth. **c)** Number of true positive (TP) insertions detected in a simulated dataset of 100 random insertions and 10 replicates, with reads of either 250bp and 1000bp and a total number of sequenced bases from 250Mb to 1.5Gb. **d)** Number of true positive (TP) insertions detected with read length of 250, 1000 and 5000 base pairs. Statistically significant differences (p<0’05) are annotated by groups with letters (a, b). **e)** Number of true positive (TP) insertions detected with read length of 100 and 350 base pairs. Statistically significant differences (p<0’05, ns = not significant).

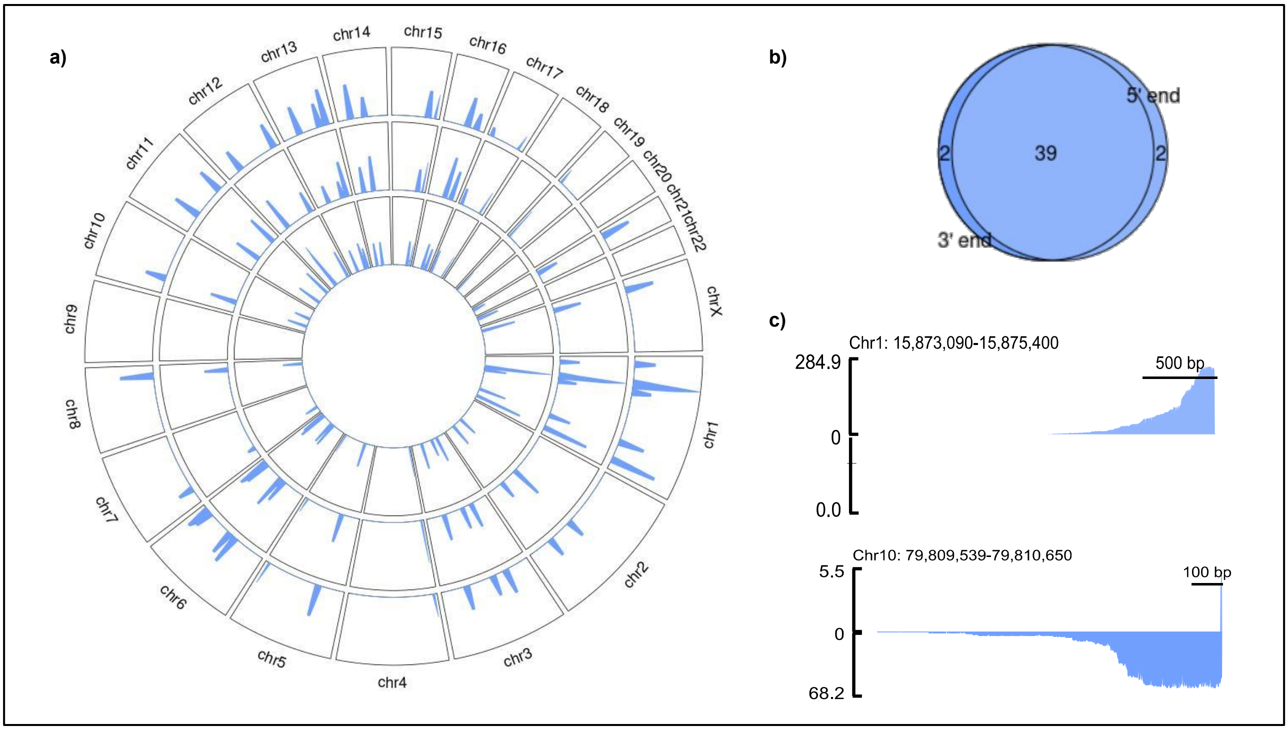

Fig S9 | Analysis of sample replicates. a) Genome-wide map displaying the overlap between insertions detected in MOPO long read insertion detection. Insertion mapping was performed to both lentiviral ends 5’ (two different replicates) and 3’. **b)** The Venn diagram summarises the overlap of common insertions with the two methods. Two insertions are found with the 5’ end mapping and not with the 3’ end while 2 new insertions are found only with the 3’ end mapping. **c)** Coverage at two selected insertion sites in the mono-clonal poli-insertional (MOPO) analyzed cell line, with 5’ end mapping (light blue) and 3’ end mapping (dark blue). Two representative insertion sites detected with only one of the end mappings are shown.

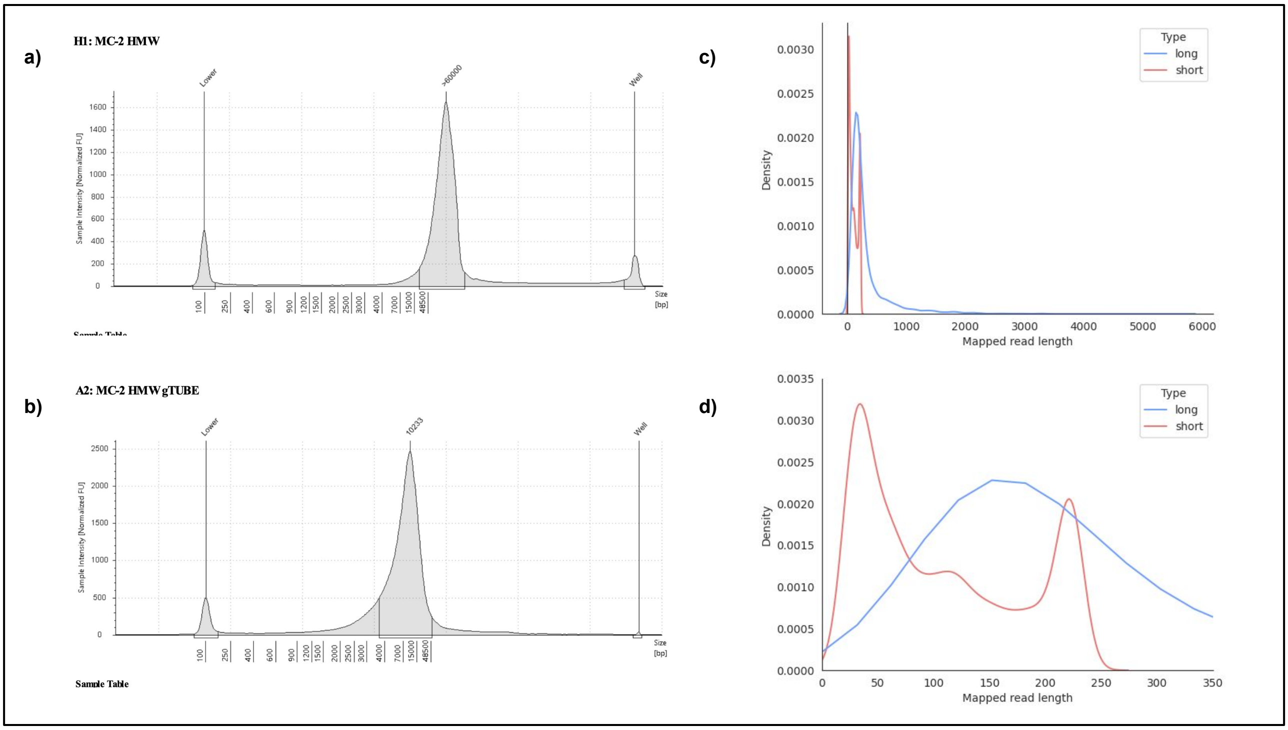

Fig S10 | Library prep impact on read length. a) Bioanalyzer showing the distribution of fragment length of the MOPO sample before random fragmentation. Mean fragment length was higher than 60 kbp. **b)** Bioanalyzer showing the distribution of fragment length of the MOPO sample after random fragmentation. Mean fragment length was ~10 kbp **c)** Density distribution of mapped read length of the MOPO sample. Mapped length of long reads range from 41 to 5718 with a mean of 329 while mapped length of short reads range from 25 to 239 with a mean of 109. **d)** Close view of the density distribution of mapped read length of the MOPO sample. With read length between 0-350.

INSERTseq protocol

Introduction

The described protocol allows retriving on/off targett patterns of a genomic perturbations of >20bp in size. Using STAT-PCR (Tsai. et al 2015) Nanopore sequencing allows for retrival of longer reads that facilitate mapping of the flanking regions of the insert by bypassing repetitive genome elements. This protocol uses ONT PCR Barcoding Kit (SQK-PBK004).

Materials

- DNA Extraction KIT
- gTUBE / sonicator
- KAPPA HYPERPREP KIT (or end repair and T4 lig NEB)
- ISA## ADAPTORS
- MISEQ comon ADAPTOR
- P05 oligos
- PBK insert specific oligos
- Rapid sequencing amplicons - four primer PCR (SQK-PSK004 or SQK-PBK004)
- LongAmp Hot Start Taq 2X Master Mix
- Thermocycler
- MiniON sequencer
- lambda Exo from NEB

Procedure

DNA extraction

1. Perform DNA extraction using either QUIAGEN COLUMN or CIRCULOMICS or column method.

Library preparation

2. Fragment DNA 35s at 12000 RPM, using 15 ug of DNA, using gTube, and based on gTUBE protocol. or use sonicator 15/15 pulse, 40% amplitude, 40-80s

3. Set up End repair using 50ul (5ug) of gTUBED DNA following above table:

End Repair

	A	B	/3	for 10	E	/3	/3*(5.5)	/3	/3*13	J
1	Component	Volume								
2	H20	0.00								
3	Fragmented, double-stranded DNA	50.00	16.67	16.67		16.67				
4	End Repair & A-Tailing Buffer*	7.00	2.33	53.67		2.33	12.83			
5	End Repair & A-Tailing Enzyme Mix*	3.00	1.00	23.00		1.00	5.50			
6	Total volume:	60.00	20.00	3.33	(per sample)	3.33	3.33	(per sample)		

4. 30 min 20ºC and 30 min 65ºC.

5. Mix Y adaptor components in a PCR tube. Adaptor sequeces for the different barcodes can be found in Annex Table 1.

6.

Y adaptor annealing:

	A	B	C
1	Component	Volume	
2	ISA## (100µM)	10.00	
3	MiSeq Common Adapter_MI (100µM)	10.00	
4	TE	80.00	
5	Total volume:	100.00	

7. Anneal Y adaptor with the following program: 95°C for 1 s; 60°C for 1s; slow ramp down (approximately -2°C/min) to 4°C; hold at 4°C. Store in -20°C.

8. Mix adaptor ligation reagents in a PCR tube, in the following order:

Y Adaptor Ligation:

	A	B	/3	/3(x4)	E	F	x3.5	H	I
1	Water	5.00	1.67	38.33	19.1666666667	2.5	17.5	1.67	21.67
2	DNA Ligation Buffer	30.00	10.00	230.00	115		105	10.00	130.00
3	DNA Ligation Mix	10.00	3.33	76.67	38.3333333333	5	35	3.33	43.33
4	ADAPTOR	5.00	1.67		#VALUE!	2.5		1.67	
5	TOTAL	50.00	16.67	15.00	in each	25	45	16.67	15.00

9. Mix by pippeting several times. Add 50 ul of ligation mixture to 60 of end prepped DNA. Incubated 15 min at 20 deg. Purify with 0.7 bead ratio using ampure XP Beads. Elute in 12

10. Set up PCR1 by mixing the following components. Pcr uses phosphorilated primers to protect from lambda exo degradation:

PCR1

	A	B	C	D	E	F	/2	/2(x10)	/2*10
1					DNA	10	12		
2					H20	14			
3					ps_P51_F	0.500	0.25	2.5	3.25
4	94	1'			ps_#####_ISP_1	1.5	0.75	7.5	9.75
5					blocking 10µM (optional,2.5 ul)			25	32.5
6	94	30"			LongAmp Hot Start Taq 2X Master Mix	25.000	12.5	125	162.5
7	61	30"	x30		TOTAL	51.000	25.5	16	12.75
8	65	8.30'						17.25	
9	65	20'							
10	4°	hold							
11									

11. Add 3 ul of lambda exo to 51 ul pcR reaction. Digested 3h at 37. 75°C for 10 min

12. used 23 ul beads for bead purification (0,75 eth)

PCR2

	A	B	C	D	E	F	/2	/2(x3.5)	I	J
1					DNA	10	5			
2					H20	11.5	5.75			
3					PBK_P52_F	0.500	0.25	2.5	3.25	1.25
4	94	1'			PBK_INSERT_GSP2__R	1.5	0.75	7.5	9.75	3.75
5					blocking 10µM (optional,2.5 ul)			25	32.5	
6	94	30"	x30		PRIMERS ONT	1.5	0.75			
7	62	30"			LongAmp Hot Start Taq 2X Master Mix	25.000	12.5	125	162.5	62.5
8	65	8.30'			TOTAL	50.000	25	16	12.75	
9	65	20'								
10	4º	hold								
11										

13. 0,5x beads were used (25ul). Elute in 11ul TE-ph8.

14. Proceed to load sample in the sequencer following PCR Barcoding *Kit* (SQK-PBK004).

15. Data analysis can be performed through a web tool or with code deposited in bitbucket and through the web page application INSERTseq.com.

Primers/adaptors:

Annex Table 1: INSERTseq ADAPTORS

	A	B
1	Miseq Common Adapter	[Phos]GATCGGAAGAGC*C*A
2	ISA01	AATGATACGGCGACCACCGAGATCTACAC AAGAAAGTTGTCGGTGTCTTTGTG TTTVVVVTTVVVVTTVVVVTTVVVVTTT ACACTCTTCACGCTCTTCCGATC*T
3	ISA02	AATGATACGGCGACCACCGAGATCTACAC TCGATTCCGTTTGTAGTCGTCTGT TTTVVVVTTVVVVTTVVVVTTVVVVTTT ACACTCTTCACGCTCTTCCGATC*T
4	ISA03	AATGATACGGCGACCACCGAGATCTACAC GAGTCTTGTGTCCCAGTTACCAGG TTTVVVVTTVVVVTTVVVVTTVVVVTTT ACACTCTTCACGCTCTTCCGATC*T
5	ISA04	AATGATACGGCGACCACCGAGATCTACAC TTCGGATTCTATCGTGTTTCCCTA TTTVVVVTTVVVVTTVVVVTTVVVVTTT ACACTCTTCACGCTCTTCCGATC*T
6	ISA05	AATGATACGGCGACCACCGAGATCTACAC CTTGTCCAGGGTTTGTGTAACCTT TTTVVVVTTVVVVTTVVVVTTVVVVTTT ACACTCTTCACGCTCTTCCGATC*T
7	ISA06	AATGATACGGCGACCACCGAGATCTACAC TTCTCGCAAAGGCAGAAAGTAGTC TTTVVVVTTVVVVTTVVVVTTVVVVTTT ACACTCTTCACGCTCTTCCGATC*T
8	ISA07	AATGATACGGCGACCACCGAGATCTACAC GTGTTACCGTGGGAATGAATCCTT TTTVVVVTTVVVVTTVVVVTTVVVVTTT ACACTCTTCACGCTCTTCCGATC*T
9	ISA08	AATGATACGGCGACCACCGAGATCTACAC TTCAGGGAACAAACCAAGTTACGT TTTVVVVTTVVVVTTVVVVTTVVVVTTT ACACTCTTCACGCTCTTCCGATC*T
10	ISA09	AATGATACGGCGACCACCGAGATCTACAC AACTAGGCACAGCGAGTCTTGGTT TTTVVVVTTVVVVTTVVVVTTVVVVTTT ACACTCTTCACGCTCTTCCGATC*T
11	ISA10	AATGATACGGCGACCACCGAGATCTACAC AAGCGTTGAAACCTTTGTCCTCTC TTTVVVVTTVVVVTTVVVVTTVVVVTTT ACACTCTTCACGCTCTTCCGATC*T

Annex Table 2: INSERTseq PCR1

	A	B	C
1	ps_P51_F	T*T*T*C*TGTTGGTGCTGATATTGC AATGATACGGCGACCACCGAGATCTACAC	
2	ps_PB_3_ISP1	T*T*C*G*CGCTATTTAGAAAGAGAG	example primer used for Piggybac payload

Annex Table 3: INSERTseq PCR2

	A	B	C
1	PBK_P52_F	TTTCTGTTGGTGCTGATATTGC AATGATACGGCGACCACCGAGATCTACAC	
2	PBK_PB3'_GSP2_R	ACTTGCCTGTCGCTCTATCTTC GAGCAATATTTCAAGAATGCATGC	example primer used for Piggybac payload

16.

17. Tsai, S. Q., Zheng, Z., Nguyen, N. T., Liebers, M., Topkar, V. V., Thapar, V., Wyvekens, N., Khayter, C., Iafrate, A. J., Le, L. P., Aryee, M. J., & Joung, J. K. (2015). GUIDE-seq enables genome-wide profiling of off-target cleavage by CRISPR-Cas nucleases. *Nature Biotechnology*, *33*(2), 187–197.
