# Supplementary material for: INSERT-seq enables high-resolution mapping of genomically integrated DNA using Nanopore sequencing
Source: Genome Biol. 2022 Oct 25;23:227. doi: 10.1186/s13059-022-02778-9 (PMC9594898; doi:10.1186/s13059-022-02778-9)
Supplement: Supplementary file 2 — Additional file 2: Supplementary tables S1-S4. [file 13059_2022_2778_MOESM2_ESM.pdf]

Table S1 - Cas9-HyPB Insertions

chromosome	start	end	coverage
chr5	179698575	179699865	14001
chr12	96582102	96582806	298
chr14	22546419	22548622	248
chr3	157115369	157116050	223
chr16	4957614	4957864	136
chr1	161531850	161531957	107
chr8	135004345	135005053	80
chr1	225555203	225555305	37
chr3	8751470	8752277	32
chr6	36444288	36444746	28
chr13	93826233	93826603	28
chr1	242746873	242747221	26
chr7	80847204	80847901	22
chr6	85564695	85565088	12
chr17	55294059	55294212	10
chr5	148681697	148681824	5
chr12	92184880	92184956	4
chr2	140344550	140344733	3
chr16	76219415	76219499	3
chr1	115811941	115812071	3
chr7	64815366	64815823	2
chr5	92337266	92337425	2
chr5	165831936	165832275	2
chr3	61645933	61646086	2
chr14	103350322	103350510	2
chr8	21963318	21963403	1
chr7	9853848	9853953	1
chr6	39567223	39567313	1
chr6	23372168	23372298	1
chr6	154838047	154838169	1
chr6	142103351	142103522	1
chr6	108304356	108304723	1
chr5	158563784	158564117	1
chr5	137841223	137842721	1
chr4	86962032	86962275	1
chr3	9724505	9724682	1
chr3	61743770	61743916	1
chr3	177344741	177344911	1
chr21	33683469	33683841	1
chr2	60533681	60533871	1
chr2	171524827	171525067	1
chr2	148456848	148457033	1
chr19	55115755	55115842	1
chr19	4246692	4246943	1
chr17	42461435	42461612	1

Table S2 - Primer sequences

PRIMERS USED FOR GUIDEseq EXPERIMENTS		
Adaptor sequence	P5_1	AATGATACGGCGACCACCGAGATCTA
	P5_2	AATGATACGGCGACCACCGAGATCTACAC
LTR	LTR3_GSP1	GGATCTCGACGCTCTCCCT tgtgactctggtaactagagatcc
	LTR3_GSP2	CCTCTCTATGGGCAGTCGGTGA tcagacccttttagtcagtgtg
PRIMERS USED FOR INSERTseq EXPERIMENTS		
target payload	primer	sequence
Adaptor sequence	ps_P51_F	T*T*T*C*TGTTGGTGCTGATATTGC AATGATACGGCGACCACCGAGATCTACAC
	PBK_P52_F	TTTCTGTTGGTGCTGATATTGC AATGATACGGCGACCACCGAGATCTACAC
LTR	ps_LTR3'_GSP1_t1	g*c*c*t*tgagtgcttcaagtagtgt
	PBK_LTR3'_GSP2_	ACTTGCCTGTCGCTCTATCTTC gtaactagagatccctcagaccctttt
PB	ps_PB_3_ISP1	T*T*C*G*CGCTATTTAGAAAGAGAG
	PBK_PB3'_GSP2_I	ACTTGCCTGTCGCTCTATCTTC GAGCAATATTTCAAGAATGCATGC
rAAV	ps_AAV_ITR_ISP1	C*C*C*T*AGTGATGGAGTTGGCCAC
	PBK_AAV_ITR_ISF	ACTTGCCTGTCGCTCTATCTTC CCACTCCCTCTCTGCGCGCTC
qPCR/ddPCR primers		
SYBR-Psi_Fw	tgaaagcgaaagggaaaccag	
SYBR-Psi_Rv	CACCCATCTCTCTCCTTCTAGCC	
SYBR-RNaseP_Fw	ggagtgaggagggatgtgaa	
SYBR-RNaseP_Rv	ATTGAGGGCACTGGAAATTG	
SYBR-AAV-GFP-F	GCCTCTTTGCACCATTCTAAAG	
SYBR-AAV-GFP-R	CAAAAGGGCCTAGCTTGGAC	

Table S3 - Unidentified insertions

Chromosome	position
chr1	108218939
chr9	41347993
chr9	61444266
chr13	4593273
chr13	7432794
chr15	16222762
chr16	41728163
chr17	18456172
chr21	4694203
chr22	12305073
chrX	60426548
chr1	149245724
chr1	222491149
chr4	51665128
chr5	49151538
chr5	181345073
chr9	41875999
chr11	52152525
chr13	610232
chr13	2168261
chr15	16802453
chr16	21899377
chr16	41907850
chr16	90243219
chrX	91544223
chrX	115824090
chr1	123802883
chr1	127967760
chr1	141347454
chr6	112816545
chr11	51840539
chr14	3668770
chr21	7036551
chr22	7997535
chrX	62211393
chrY	38970872
chrY	51364199
chrY	52162792
chr2	91015069
chr2	96019862
chr8	77548
chr9	44799078
chr9	60352836
chr15	14197174
chr21	1881234
chrX	52941260
chrY	37617613

Table S3 - Unidentified insertions

chrY	52255224
chr1	127680019
chr3	92353009
chr16	15202776
chr16	42643590
chr17	47481890
chr18	15305063
chr21	8753930
chrX	92841383
chr1	138005042
chr1	138084966
chr15	18410856
chr16	37535769
chr22	14158363
chrX	30390
chrY	33560302
chrY	37438884
chrY	50544075
chr1	131663063
chr14	15131023
chr18	17159518
chr22	3212148
chr22	6488733
chrY	24939708
chr1	129862871
chr6	58677426
chr9	46265678
chr13	14557939
chr14	11221294
chr14	19045841
chr15	6423420
chr16	90200883
chr17	46667816
chrX	61363867
chrY	46599178
chr2	87175946
chr9	43398688
chr13	113700952
chr14	373771
chr15	5406639
chr22	9915341
chr1	13305867
chr2	175486317
chr9	44479515
chr10	50103603
chr13	2465844
chr13	7011404
chr14	14466289

Table S3 - Unidentified insertions

chr15	21565559
chr17	18405528
chr21	1504519
chr21	11721287
chrX	107159080
chrX	153292669

Table S4 - MOPO sample lentiviral integrations

MOPO 5'					MOPO 3' replicate					MOPO 5' replicate					MOPO GS all reads					MOPO GS subset				
Chromosome	Start	End	Coverage	RSS	Chromosome	Start	End	Coverage	RSS	Chromosome	Start	End	Coverage	RSS	Chromosome	Start	End	Coverage	RSS	Chromosome	Start	End	Coverage	RSS
chr1	15873090	15875400	143	0.001336						chr1	15874259	15875385	259	0.000789	chr1	15875163	15875383	2489	2.00E-05	chr1	15875163	15875383	324	0.000626
chr1	59843135	59846088	101	0.009731	chr1	59846081	59847682	580	0.000602	chr1	59844882	59846092	250	0.002678	chr1	59845861	59846088	2463	1.80E-05	chr1	59845864	59846088	338	0.000883
chr1	62598746	62601365	106	0.008995	chr1	62601357	62602801	485	0.00052	chr1	62600094	62601365	323	0.000665	chr1	62601140	62601365	2376	2.00E-05	chr1	62601140	62601364	313	0.001291
chr1	63568919	63570854	126	0.004137	chr1	63570836	63572482	628	0.000303	chr1	63569397	63570845	234	0.003241	chr1	63570617	63570842	2286	1.80E-05	chr1	63570617	63570842	319	0.000917
chr1	77210774	77212894	84	0.014051	chr1	77209272	77210788	519	0.000609	chr1	77210774	77211840	241	0.001379	chr1	77210781	77211006	1977	2.90E-05	chr1	77210782	77211005	255	0.002327
chr1	188551355	188554270	85	0.010381	chr1	188549470	188551375	740	0.000532	chr1	188551354	188552633	157	0.007354	chr1	188551354	188551579	1992	2.00E-05	chr1	188551355	188551579	290	0.001338
chr1	227749374	227752406	104	0.013323	chr1	227748550	227750269	621	0.000297	chr1	227749370	227750727	274	0.001068										
chr1	229319899	229322075	81	0.017682	chr1	229318327	229319907	708	0.000239	chr1	229319897	229321221	237	0.001673	chr1	229319899	229320126	2095	2.20E-05	chr1	229319899	229320126	290	0.000749
chr10	33269439	33271731	111	0.011446	chr10	33271716	33273100	546	0.00011	chr10	33270181	33271731	227	0.002487	chr10	33271498	33271725	2405	2.30E-05	chr10	33271501	33271724	311	0.001717
					chr10	79809539	79810650	62	0.089338															
chr11	43382440	43385505	104	0.010537	chr11	43380764	43382452	474	0.001022	chr11	43382440	43384001	245	0.004648	chr11	43382439	43382664	1684	4.00E-05	chr11	43382439	43382663	230	0.002136
chr11	113257668	113260583	57	0.13489	chr11	113255719	113257674	656	0.000589	chr11	113257665	113259435	258	0.004438	chr11	113257667	113257891	1456	6.30E-05	chr11	113257667	113257891	217	0.002851
chr12	30650720	30653245	82	0.018093	chr12	30648406	30650730	631	0.00151	chr12	30650061	30652049	240	0.005994	chr12	30650723	30650948	2103	2.40E-05	chr12	30650724	30650947	259	0.002136
chr12	118363938	118366784	71	0.083025	chr12	118362215	118364164	491	0.00154	chr12	118363939	118365222	123	0.009723	chr12	118363941	118364169	1934	2.30E-05	chr12	118363942	118364169	240	0.002575
chr13	37012216	37015426	138	0.005372	chr13	37015416	37017001	722	0.000349	chr13	37014378	37015428	339	0.000581	chr13	37015200	37015426	2252	2.00E-05	chr13	37015201	37015426	308	0.000914
chr13	82126272	82128937	57	0.227379	chr13	82128910	82130746	574	0.000612	chr13	82127955	82128940	263	0.002734	chr13	82128703	82128928	2006	2.30E-05	chr13	82128704	82128927	271	0.002933
chr13	95581825	95585866	114	0.029002	chr13	95580440	95581838	612	0.00016	chr13	95581822	95583903	329	0.003043	chr13	95581826	95582051	1994	3.20E-05	chr13	95581827	95582050	245	0.003364
chr14	27861486	27865329	64	0.040549	chr14	27865321	27866902	357	0.000806	chr14	27863854	27865333	146	0.004365	chr14	27865104	27865330	1242	0.000112	chr14	27865106	27865329	162	0.011038
chr14	59870506	59872712	83	0.006646	chr14	59872695	59874292	558	0.000553	chr14	59870681	59872711	210	0.009066	chr14	59872481	59872708	2057	3.30E-05	chr14	59872484	59872708	293	0.001668
chr15	78588158	78591117	111	0.016459	chr15	78586518	78588181	787	9.80E-05	chr15	78588161	78589442	325	0.001529	chr15	78588161	78588386	2184	2.80E-05	chr15	78588161	78588386	288	0.001582
chr15	99327982	99330675	165	0.005045	chr15	99330664	99332125	459	0.000614	chr15	99328864	99330672	385	0.003003	chr15	99330447	99330672	2725	1.30E-05	chr15	99330448	99330671	366	0.001151
chr16	53233002	53236321	116	0.009737	chr16	53236314	53238108	482	0.001023	chr16	53234473	53236891	450	0.001539	chr16	53236096	53236321	2799	1.60E-05	chr16	53236097	53236321	361	0.000943
chr16	71779733	71781578	104	0.005715	chr16	71781570	71783084	593	0.000761	chr16	71780043	71781754	368	0.001155	chr16	71781349	71781576	2734	1.40E-05	chr16	71781353	71781576	340	0.001651
chr17	7883412	7884385	60	0.01775	chr17	7881804	7883418	464	0.000826	chr17	7883411	7884364	233	0.001144	chr17	7883411	7883635	1614	3.90E-05	chr17	7883411	7883635	226	0.002117
chr17	77959376	77961103	92	0.003697	chr17	77961097	77962794	301	0.001155	chr17	77959679	77961104	266	0.000751	chr17	77960877	77961103	2129	3.00E-05	chr17	77960877	77961103	292	0.001865
chr19	1124993	1126851	83	0.010737	chr19	1126821	1128476	524	0.000458	chr19	1125767	1127738	248	0.007785	chr19	1126603	1126828	1887	2.40E-05	chr19	1126604	1126828	275	0.002283
chr2	130810788	130812904	61	0.022393	chr2	130812893	130814262	330	0.001329	chr2	130811025	130812906	181	0.007126	chr2	130812509	130812905	1673	0.000176	chr2	130812681	130812904	210	0.009702
chr2	188059521	188061692	79	0.00621	chr2	188061673	188063268	369	0.00116	chr2	188060640	188061692	136	0.003168	chr2	188061466	188061691	1305	7.90E-05	chr2	188061468	188061691	169	0.005862
chr21	14268490	14271744	85	0.030428	chr21	14267690	14268592	267	0.006577	chr21	14268490	14269728	205	0.001739	chr21	14268491	14268716	1887	3.00E-05	chr21	14268491	14268714	252	0.002325
					chr22	11066754	11068241	45	0.050116															
chr3	52592419	52594998	122	0.005011	chr3	52594985	52596800	644	0.000694	chr3	52593534	52595005	448	0.000365	chr3	52594771	52594997	2581	2.00E-05	chr3	52594773	52594997	336	0.001126
chr3	83387428	83389725	73	0.007486	chr3	83389714	83390891	430	0.000464	chr3	83388448	83389722	266	0.002619	chr3	83389496	83389720	1676	4.80E-05	chr3	83389497	83389720	214	0.005204
chr3	144120580	144123289	125	0.01417	chr3	144123280	144125069	905	0.000244	chr3	144121768	144123289	488	0.000781	chr3	144123059	144123288	2701	2.80E-05	chr3	144123061	144123288	371	0.001232
chr4	1818333	1820923	40	0.085581	chr4	1816682	1818774	652	0.00107	chr4	1817866	1820372	131	0.036583										
chr5	55710126	55713662	47	0.055292	chr5	55708472	55710138	201	0.004904	chr5	55710126	55711799	172	0.004295	chr5	55710126	55710356	1866	3.30E-05	chr5	55710126	55710356	273	0.001547
chr5	179883252	179885869	38	0.136193	chr5	179881900	179883259	135	0.011939	chr5	179883253	179884136	31	0.450602	chr5	179883252	179883477	518	0.000409	chr5	179883253	179883476	72	0.03455
chr6	72363638	72366947	120	0.006347	chr6	72366933	72368640	497	0.000945	chr6	72365567	72366950	205	0.004025	chr6	72366720	72366945	2155	2.20E-05	chr6	72366721	72366945	292	0.001278
chr6	88853881	88856667	63	0.027774	chr6	88852276	88853902	508	0.00055	chr6	88853890	88855856	141	0.00685	chr6	88853889	88854118	1192	4.20E-05	chr6	88853890	88854118	137	0.004814
chr6	149495989	149498027	102	0.008525	chr6	149497997	149500013	757	0.000279	chr6	149496548	149498022	223	0.002598	chr6	149497795	149498019	1754	3.60E-05	chr6	149497795	149498018	246	0.002395
chr6	156758635	156761366	90	0.020233	chr6	156761353	156763036	622	0.001245	chr6	156759548	156761368	243	0.005517	chr6	156761142	156761366	2103	3.10E-05	chr6	156761143	156761366	299	0.0024
chr7	32733447	32735168	31	0.403716						chr7	32733447	32733907	78	0.038251	chr7	32733450	32733675	52	0.093922					
chr8	124366563	124370261	148	0.008507	chr8	124365233	124366572	635	0.000118	chr8	124366563	124367918	372	0.000717	chr8	124366563	124366781	3136	1.10E-05	chr8	124366564	124366781	419	0.000621
chrX	24876961	24880093	102	0.018245	chrX	24874621	24876968	743	0.000701	chrX	24876961	24878575	398	0.001018	chrX	24876961	24877186	2315	1.20E-05	chrX	24876961	24877185	284	0.001617
